# Investigating the causal effects of physical activity and sedentary behavior on hernia risk: A two-sample Mendelian randomization approach

**DOI:** 10.1097/MD.0000000000050555

**Published:** 2026-09-04

**Authors:** Qian Yu, Li Wang, Rujun Zhai, Guodong Song

**Affiliations:** a Department of Gastrointestinal Surgery, The Second Hospital of Tianjin Medical University, Tianjin, People’s Republic of China.

**Keywords:** hernia, inguinal hernia, Mendelian randomization, physical activity, sedentary behavior

## Abstract

The global prevalence of hernia is increasing, particularly due to the aging population. Although physical activity and sedentary behavior are known to influence various health outcomes, their specific roles in hernia development remain inadequately investigated. This study aimed to systematically assess the causal relationships between physical activity, sedentary behavior, and the risk of hernia development using Mendelian randomization (MR) analysis. We used genome-wide association study data from the UK Biobank and FinnGen Biobank to explore the causal effects of sedentary behavior on hernia risk via 5 distinct MR approaches. To ensure the reliability of our risk models, we performed sensitivity analyses, including Cochran’s *Q* test, MR-Egger intercept analysis, leave-one-out analysis, and funnel plots. Furthermore, we employed multivariable MR analysis to determine the independent causal effects of both physical activity and sedentary behavior. Our findings indicate that moderate-to-vigorous physical activity (MVPA) was significantly associated with an increased risk of inguinal hernia, with an odds ratio of 1.844 (95% confidence interval, 1.181–2.879; *P* = .007). Associations between sedentary behavior and diaphragmatic or umbilical hernia were inconsistent across methods and were sensitive to pleiotropy; thus, no robust causal relationship was established. Multivariable MR analysis further confirmed the independent and significant causal relationship between MVPA and inguinal hernia, with an odds ratio of 1.901 (95% confidence interval, 1.098–3.291; *P* = .022). Our study highlights a significant association between MVPA and an increased risk of inguinal hernia, suggesting that physical activity should be carefully considered in preventive strategies for hernias. However, the relationship between sedentary behavior and hernia development warrants further investigation.

## 1. Introduction

Hernia is a common condition worldwide, with inguinal hernia being the most prevalent type, especially among males. As the global population ages, the incidence of hernias is also on the rise.^[1]^ While the mechanisms underlying hernia development and its associated risk factors have been extensively studied, several aspects remain unresolved. Clinical and epidemiological studies have shown that factors such as obesity, chronic cough, constipation, and heavy physical labor may increase intra-abdominal pressure, thereby contributing to the development of hernia.^[2–4]^ However, there is still no clear consensus on the exact relationship between physical activity, sedentary behavior, and hernia risk. The discrepancy in findings may be attributed to differences in study methodologies and population samples.

Mendelian randomization (MR), a research method that leverages genetic variations to explore causal relationships, offers a novel approach for understanding the risk factors for hernia development.^[5]^ Unlike traditional observational studies, MR minimizes the confounding effects of environmental factors and lifestyle choices, thereby providing a more robust evaluation of causal relationships. Using genetic variants as instrumental variables, MR effectively reduces the biases associated with reverse causality, making it an important tool in modern epidemiological research. This approach allows researchers to more accurately assess whether factors such as physical activity and sedentary behavior have a true causal effect on hernia risk, rather than merely being correlated.

The study aimed to investigate the causal effects of physical activity and sedentary behavior on hernia risk using a two-sample MR approach. By employing this innovative method, we hope to provide more reliable evidence regarding the potential influence of lifestyle factors on hernia development, which could inform preventive and interventional strategies for hernia management.

## 2. Materials and methods

### 2.1. Study design and data sources

This study utilizes a two-sample MR analysis to examine the causal relationships between physical activity, sedentary behavior, and hernia occurrence. In this analysis, physical activity and sedentary behavior serve as exposure variables, while hernia serves as the outcome variable, with single nucleotide polymorphisms (SNPs) functioning as instrumental variables. To qualify these SNPs as valid instrumental variables, they must meet specific criteria: they should exhibit a robust correlation with the exposure variables, remain independent of confounding factors, and not directly affect the outcome variable.^[6]^ The selection of these instrumental variables followed a systematic process consisting of several steps: first, SNPs with *P* > 5 × 10^−8^ from genome-wide association studies (GWAS) were excluded; second, SNPs exhibiting linkage disequilibrium (*r*^2^ > 0.001) were eliminated; third, SNPs associated with known confounding factors were discarded; and finally, incompatible alleles and palindromic SNPs were excluded. The validity of the instrumental variables is assessed using the *F*-statistic (β^2^/SE^2^, where SE = standard error), with SNPs yielding an *F*-statistic below 10 considered “weak instrumental variables” and subsequently excluded from the analysis.^[7]^ All analyses were performed in accordance with the Strengthening the Reporting of Observational Studies in Epidemiology using MR guidelines.^[8]^

Genetic data on physical activity and sedentary behavior were obtained from the UK Biobank, a comprehensive health resource offering valuable insights into a range of health factors. Physical activity was assessed via self-reported moderate-to-vigorous physical activity (MVPA, involving approximately 377,000 participants) and accelerometer-based average activity (ABAA, involving approximately 91,000 participants).^[9,10]^ To estimate physical activity levels, the total weekly minutes spent on moderate-intensity activities, such as light weight lifting or moderate cycling, were multiplied by 4, while those dedicated to vigorous activities, such as rapid cycling or intense weightlifting, were multiplied by 8 to calculate metabolic equivalent values.^[9]^ Machine learning algorithms were employed to analyze the accelerometer data, facilitating the estimation of participants’ average activity levels.^[10]^ In this analysis, 18 SNPs associated with MVPA and 8 SNPs linked to ABAA were identified as instrumental variables (Table S1, Supplemental Digital Content 1). In addition, summary GWAS data from the UK Biobank provided instrumental variables for 3 types of sedentary behavior – television watching, computer use, and driving—yielding 108, 77, and 7 SNPs remaining after filtration for each behavior, respectively (Table S1, Supplemental Digital Content 1).^[11]^

The hernia data were sourced from the FinnGen Biobank, which includes multiple types of hernia.^[12]^ The primary hernia categories investigated included overall hernia (28,235 cases and 190,557 controls), inguinal hernia (17,096 cases and 190,557 controls), diaphragmatic hernia (5741 cases and 190,557 controls), femoral hernia (700 cases and 190,557 controls), umbilical hernia (4224 cases and 190,557 controls), and unspecified abdominal hernia (517 cases and 190,557 controls).

### 2.2. MR analysis

This investigation employed 5 distinct methods to assess causal effects using MR analysis: weighted median, weighted mode, inverse variance weighted (IVW), MR-Egger, and simple mode. The IVW method is the primary analytical approach that provides the most accurate effect estimates when all instrumental variables are valid.^[13]^ The weighted median method can yield reliable effect estimates even when at least 50% of the instrumental variables are invalid.^[14]^ The MR-Egger method plays a crucial role in detecting violations of instrumental variable assumptions and facilitates valid causal conclusions in the presence of such violations.^[15]^

### 2.3. Sensitivity analysis

Several sensitivity analyses were conducted to ensure the robustness of the findings. Cochran’s *Q* test was used to assess heterogeneity among the effects of the instrumental variables. A *P* < .05 indicates significant heterogeneity, necessitating the use of the IVW random effects model, whereas the absence of heterogeneity leads to the application of the IVW fixed effects model.^[16]^ The MR-Egger intercept test was conducted to explore pleiotropy, examining whether specific SNPs influenced causal effect estimates through associations with other phenotypes. If horizontal pleiotropy is identified, conclusions can be drawn based on the MR-Egger methodology.^[17]^ In addition, the Steiger test was employed to assess whether the causal relationship might be influenced by reverse causation.^[18]^

Visual aids, such as scatter plots, illustrate the relationship between exposure and outcome, whereas forest plots depict the contributing factors. The contribution of each instrumental variable to the overall effect was assessed using leave-one-out analysis, which examines how the exclusion of specific SNPs affects the overall effect estimate.^[19]^

### 2.4. Multivariable Mendelian randomization

Multivariable Mendelian randomization (MVMR) allows for simultaneous assessment of the causal effects of multiple factors on outcome risks by integrating them into a unified model.^[20]^ In this study, we considered both physical activity and sedentary behavior owing to their strong association, with the aim of evaluating whether each exposure has an independent effect on the outcome.

### 2.5. Statistical methods

Findings concerning causal effects are presented as odds ratios (ORs) with corresponding 95% confidence intervals (CIs). All statistical analyses were performed using 2-sided tests, with *P*  < .05 considered statistically significant. The analyses were conducted using R software (version 4.3.2; R Core Team), incorporating relevant packages such as TwoSampleMR (version 0.6.7; University of Bristol) and MVMR (version 0.4.0; University of Bristol).

## 3. Results

### 3.1. MR estimates

The *F*-statistics for all selected SNPs were>10, indicating that weak instrument bias was unlikely to be a concern (see Table S1, Supplemental Digital Content 1 for details). Table 1 presents the causal relationships between physical activity and sedentary behavior in relation to different types of hernia, assessed using the MR-Egger, weighted median, and IVW methodologies (Table 1). Primary IVW analysis identified a significant association between MVPA and an increased risk of inguinal hernia, with an OR of 1.844 (95% CI, 1.181–2.879; *P* = .007). Although the MR-Egger and weighted median methods did not yield statistically significant results, a consistent trend indicating an elevated risk was observed across all 3 methodologies. In addition, the IVW analysis revealed that prolonged television viewing was associated with an increased risk of diaphragmatic hernia (OR = 1.468; 95% CI, 1.006–2.141; *P* = .046), although this finding was not corroborated by the MR-Egger and weighted median methods. Furthermore, the MR-Egger analysis indicated an association between the duration of television viewing and an increased risk of umbilical hernia (OR = 31.175; 95% CI, 2.822–344.372; *P* = .006); however, the IVW and weighted median methods could not replicate this. Subsequent analyses revealed that prolonged computer use was associated with a decreased risk of diaphragmatic hernia (OR = 0.052; 95% CI, 0.005–0.492; *P* = .012), although the other 2 methodologies did not yield statistically significant results. The remaining results did not show any significant association.

**Table 1:** MR assessment of the causal relationship between physical activity, sedentary behavior, and hernia.

Exposure	Outcome	MR-Egger		Weighted median		IVW	
		OR (95% CI)	*P*	OR (95% CI)	*P*	OR (95% CI)	*P*
MVPA	Hernia	0.969 (0.060–15.764)	.983	1.386 (0.827–2.323)	.216	1.584 (0.981–2.560)	.060
	Inguinal hernia	3.292 (0.264–41.136)	.369	1.739 (0.915–3.303)	.091	**1.844 (1.181–2.879**)	**.007**
	Diaphragmatic hernia	0.081 (0.001–13.121)	.348	0.826 (0.286–2.388)	.725	1.015 (0.415–2.481)	.974
	Femoral hernia	6.815 (1.40E-04–3.32E + 05)	.732	1.105 (0.082–14.960)	.940	0.614 (0.092–4.110)	.615
	Umbilical hernia	3.863 (0.026–582.113)	.605	1.555 (0.517–4.674)	.432	2.081 (0.880–4.922)	.095
	Unspecified abdominal hernia	2.786 (9.98E-06–7.86E + 05)	.875	0.599 (0.032–11.366)	.733	0.602 (0.066–5.472)	.652
ABAA	Hernia	0.896 (0.667–1.203)	.491	0.980 (0.927–1.036)	.472	0.987 (0.927–1.051)	.686
	Inguinal hernia	0.870 (0.624–1.212)	.441	0.996 (0.930–1.066)	.901	1.010 (0.939–1.086)	.793
	Diaphragmatic hernia	1.000 (0.729–1.372)	.999	0.955 (0.877–1.040)	.287	0.967 (0.901–1.038)	.350
	Femoral hernia	1.262 (0.529–3.011)	.619	0.894 (0.701–1.139)	.364	0.860 (0.709–1.043)	.126
	Umbilical hernia	0.939 (0.583–1.514)	.806	0.970 (0.866–1.087)	.600	0.917 (0.831–1.012)	.084
	Unspecified abdominal hernia	1.734 (0.530–5.675)	.398	0.831 (0.608–1.136)	.246	0.902 (0.689–1.180)	.452
Television watching	Hernia	0.826 (0.323–2.111)	.691	1.175 (0.897–1.538)	.242	1.098 (0.907–1.329)	.338
	Inguinal hernia	0.443 (0.125–1.570)	.210	1.163 (0.821–1.648)	.396	0.988 (0.764–1.279)	.929
	Diaphragmatic hernia	0.488 (0.076–3.125)	.451	1.292 (0.750–2.227)	.356	**1.468 (1.006–2.141**)	**.046**
	Femoral hernia	1.100 (0.007–172.339)	.971	2.586 (0.594–11.250)	.205	1.152 (0.412–3.218)	.787
	Umbilical hernia	**31.175 (2.822–344.372**)	**.006**	1.791 (0.898–3.574)	.098	1.505 (0.912–2.483)	.109
	Unspecified abdominal hernia	0.073 (0.000–43.169)	.424	0.335 (0.054–2.091)	.242	0.549 (0.151–1.999)	.363
Computer use	Hernia	0.442 (0.112–1.737)	.246	0.977 (0.721–1.322)	.878	1.022 (0.780–1.305)	.863
	Inguinal hernia	0.667 (0.117–3.812)	.651	1.035 (0.698–1.534)	0.865	1.096 (0.804–1.493)	.562
	Diaphragmatic hernia	**0.052 (0.005–0.492**)	**.012**	0.717 (0.396–1.296)	.271	0.775 (0.515–1.167)	.222
	Femoral hernia	0.068 (0.000–42.962)	.417	1.233 (0.243–6.252)	.800	1.657 (0.524–5.233)	.390
	Umbilical hernia	2.971 (0.220–40.125)	.415	1.413 (0.721–2.769)	.314	1.070 (0.673–1.700)	.776
	Unspecified abdominal hernia	0.001 (2.51E-07–5.915)	.126	1.087 (0.142–8.315)	.936	1.426 (0.308–6.596)	.650
Driving	Hernia	1773.117 (0.029–1.08E + 08)	.254	1.167 (0.431–3.160)	.761	0.723 (0.208–2.509)	.609
	Inguinal hernia	62,304.678 (0.272–1.43E + 10)	.154	0.355 (0.095–1.332)	.125	0.445 (0.092–2.148)	.314
	Diaphragmatic hernia	0.718 (2.56E-07–2.01E + 06)	.967	0.512 (0.077–3.396)	.488	0.432 (0.100–1.860)	.260
	Femoral hernia	0.058 (1.24E-18–2.72E + 15)	.892	0.398 (0.004–43.163)	.700	0.388 (0.007–20.860)	.642
	Umbilical hernia	1.218 (2.79E-10–5.31E + 09)	.987	2.084 (0.238–18.285)	.507	3.247 (0.415–25.423)	.262
	Unspecified abdominal hernia	3.07E + 07(4.04E-16–2.33E + 30)	.556	0.461 (0.001–223.112)	.806	0.096 (0.001–17.324)	.376

Bold values indicate statistically-significant associations (*P* < .05).

ABAA = accelerometer-based average activity, CI = confidence interval, IVW = inverse variance weighted, MR = Mendelian randomization, MVPA = moderate-to-vigorous physical activity, OR = odds ratio.

### 3.2. Sensitivity analysis

In the assessment of heterogeneity, several factors exhibited varying degrees of impact on the incidence of hernias. Specifically, MVPA demonstrated variability in both the overall hernia incidence and diaphragmatic hernia. Similarly, the association between active behavioral assessment and both overall hernia incidence and inguinal hernia demonstrated heterogeneity. The amount of time spent watching television was found to be heterogeneous with respect to umbilical hernia, while the duration of computer usage revealed heterogeneity concerning the overall hernia incidence, inguinal hernia, and other types of abdominal hernia. In addition, the time spent driving demonstrated variability in relation to the overall hernia incidence and inguinal hernia, with all findings being statistically significant (*P* < .05; Table 2; Table S2, Supplemental Digital Content 2). The study employed the IVW random effects model to derive causal estimates; however, the results were not statistically significant, which did not affect the overall analysis. The MR-Egger intercept test indicated the presence of horizontal pleiotropy in the relationships between television viewing duration and umbilical hernia, as well as between computer usage duration and diaphragmatic hernia. In contrast, no other associations showed signs of horizontal pleiotropy (*P* > .05; Table 2; Table S2, Supplemental Digital Content 2). The MR-Egger method yielded statistically significant results in cases where horizontal pleiotropy was present. Furthermore, the Steiger test examined the possibility of reverse causation bias, finding no evidence of such bias between MVPA and inguinal hernia, nor between television viewing duration and umbilical hernia (*P* < .05; Table 2). However, potential reverse causation bias was indicated between television viewing duration and diaphragmatic hernia, as well as between computer usage duration and diaphragmatic hernia (*P* > .05). Scatter plots effectively illustrate the effect sizes associated with each MR method used in this study.

**Table 2:** Sensitivity analysis of the causal associations between physical activity, sedentary behavior, and hernia.

Exposure	Outcome	Heterogeneity test			Pleiotropic test		Steiger test	
		Method	*Q*	*P*	Egger intercept	*P*	Direction	*P*
MVPA	Inguinal hernia	MR-Egger	15.452	.492	−0.009	.654	True	.014
		IVW	15.661	.548				
Television watching	Diaphragmatic hernia	MR-Egger	101.951	.511	0.013	.238	False	.653
		IVW	103.359	.499				
Television watching	Umbilical hernia	MR-Egger	130.016	.037	−0.036	.013	True	.017
		IVW	138.067	.014				
Computer use	Diaphragmatic Hernia	MR-Egger	71.720	.553	0.035	.019	False	.065
		IVW	77.450	.400				

IVW = inverse variance weighted, MR = Mendelian randomization, MVPA = moderate-to-vigorous physical activity.

**Figure 1.**
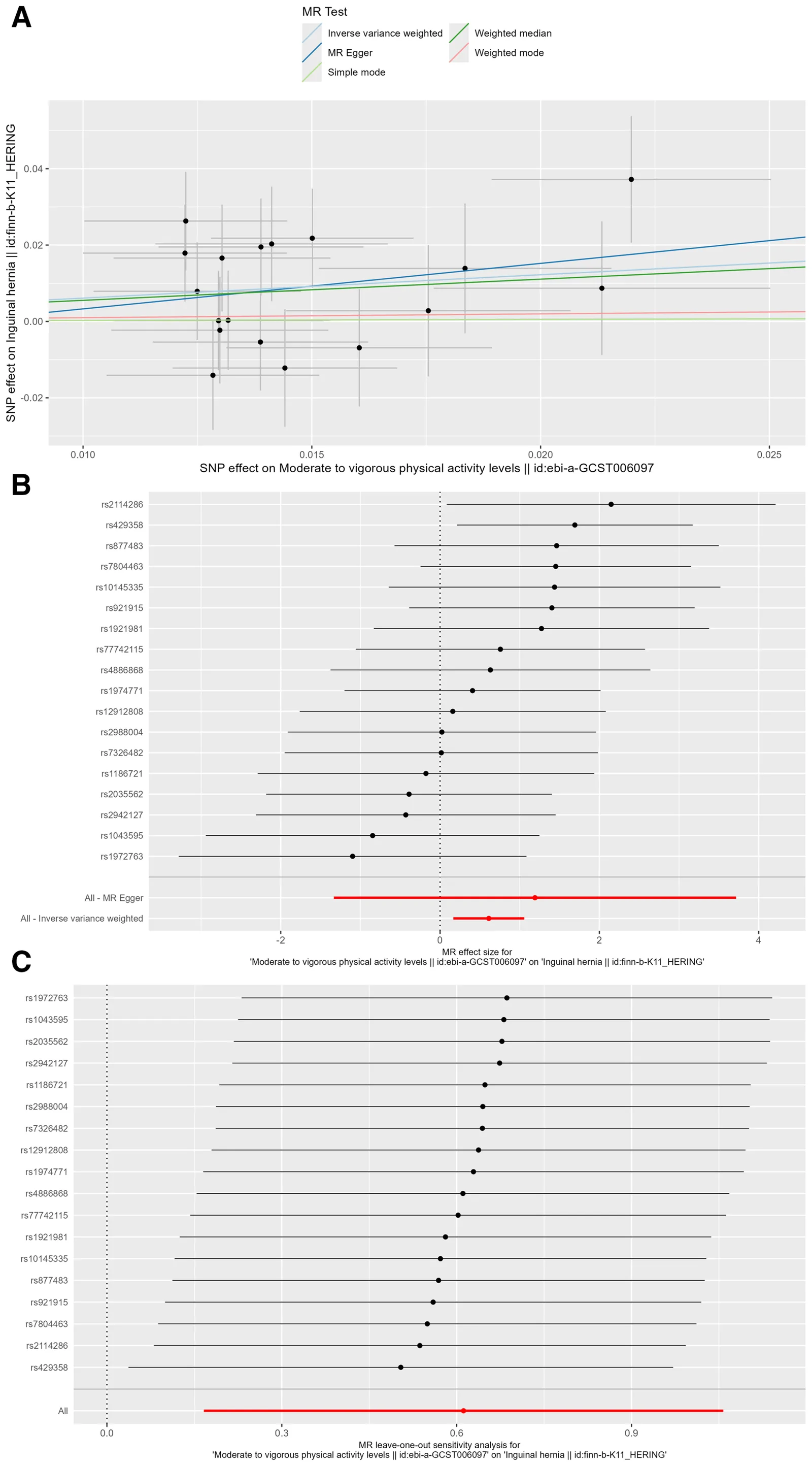
Causal inference diagram of MVPA and inguinal hernia: (A) scatter plot; (B) forest plots; (C) leave-one-out analysis. MR = Mendelian randomization, MVPA = moderate-to-vigorous physical activity, SNP = single nucleotide polymorphism.

In addition, the scatter plot illustrates the effect sizes of each MR method used in this study (Fig. 1A; Fig. S1, Supplemental Digital Content 3). Forest plots demonstrated that individual SNPs did not significantly influence the overall findings (Fig. 1B; Fig. S2, Supplemental Digital Content 4). The leave-one-out analysis further confirmed this, as the sequential removal of individual SNPs yielded results consistent with the primary MR findings, with no outliers detected throughout the analysis (Fig. 1C; Fig. S3, Supplemental Digital Content 5).

### 3.3. MVMR analysis

**Figure 2.**
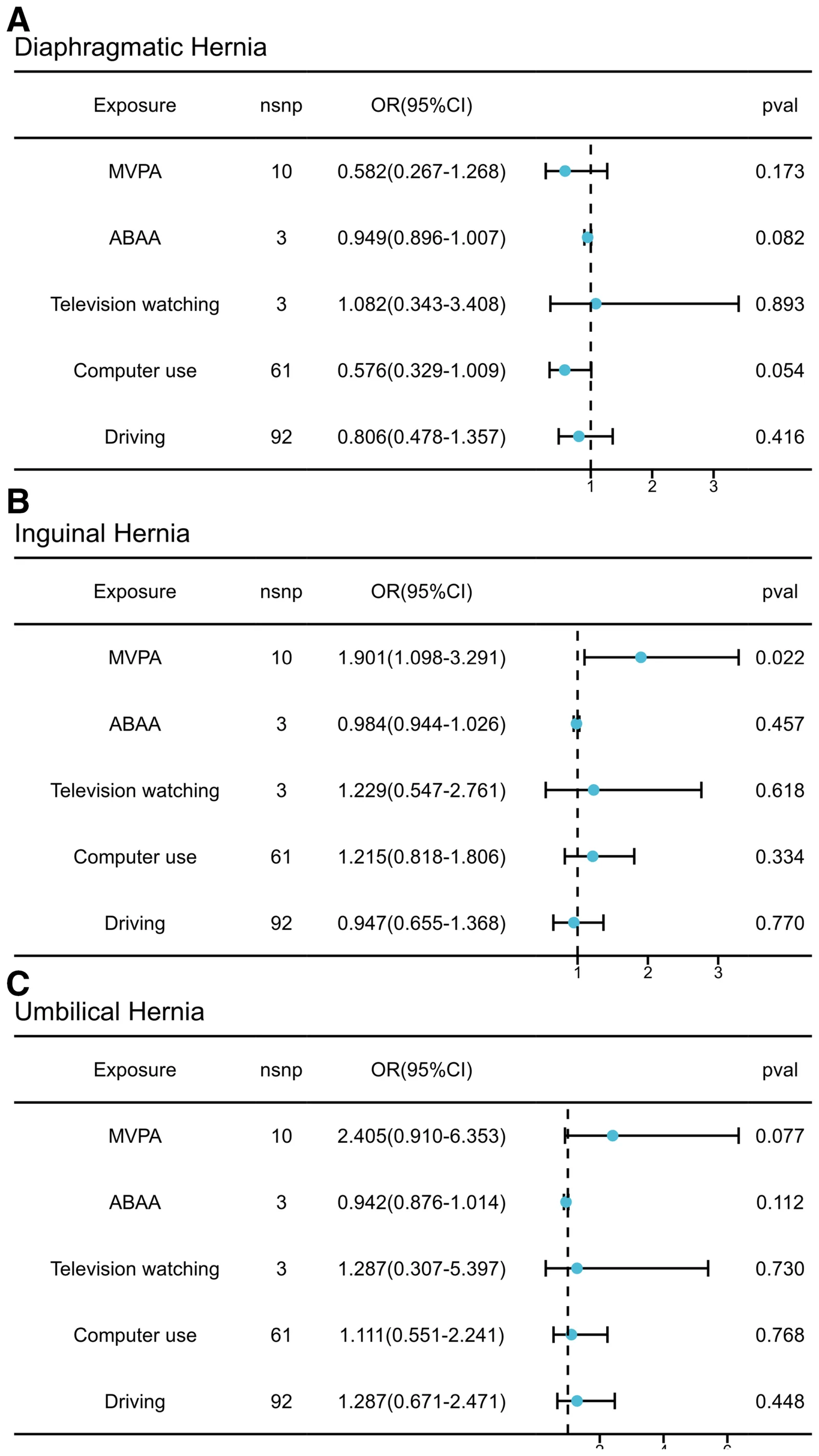
Multivariable MR analysis of inguinal hernia, diaphragmatic hernia, and umbilical hernia. ABAA = accelerometer-based average activity, CI = confidence interval, MVPA = moderate-to-vigorous physical activity, MR = Mendelian randomization, nsnp = number of single‑nucleotide polymorphisms, OR = odds ratio.

Given the established association between physical activity and sedentary behavior, this study conducted MVMR analysis, focusing on inguinal, diaphragmatic, and umbilical hernia. This study aimed to assess the independent causal relationships between different exposure factors and the development of hernias. The results revealed that only MVPA had an independent causal effect on inguinal hernia, with an OR of 1.901 (95% CI, 1.098–3.291; *P* = .022; Fig. 2). In contrast, no independent causal associations were identified for diaphragmatic or umbilical hernias (Fig. 2).

## 4. Discussion

The present investigation employed a two-sample MR approach, integrating data from the UK Biobank and comprehensive GWAS data from the FinnGen Biobank to examine potential causal relationships between physical activity, sedentary behavior, and the incidence of hernia. The results suggest that MVPA is associated with an increased risk of inguinal hernia. The associations between television watching and umbilical hernia, and between computer use and diaphragmatic hernia, were driven by methods sensitive to violations of MR assumptions and were not supported by more robust methods like IVW or weighted median. Given the implausible effect size for television watching on umbilical hernia and the contradictory directions for diaphragmatic hernia, caution is warranted. These findings may reflect bias from weak instruments or pleiotropy and should not be considered evidence of a causal link. A considerable body of research has employed MR methodologies to assess the impact of physical activity and sedentary behavior on various health outcomes. For instance, studies in the gastrointestinal field have demonstrated that sedentary behavior is associated with an increased risk of gastroesophageal reflux, gastric ulcers, and duodenal ulcers, whereas physical activity appears to lower the incidence of these conditions.^[21]^ In the field of mental health and behavioral studies, physical activity has been associated with a reduced risk of depression and smoking, whereas sedentary behavior has been linked to an increased risk of anorexia and schizophrenia.^[22]^ Moreover, the existing literature has established clear causal relationships between leisure screen time, physical activity, and the incidence of migraines, pulmonary fibrosis, and osteoarthritis.^[23–25]^ This study represents the first effort to investigate the potential causal links between physical activity and sedentary behavior in relation to hernia occurrence using MR, thereby contributing to the understanding of how these behaviors influence health.

Research on the relationship between physical activity, sedentary behavior, and hernia remains limited. Previous studies have indicated that engaging in vigorous physical activity may increase the risk of developing inguinal hernia, particularly among individuals involved in heavy physical labor. For example, an observational study conducted in the United Kingdom found a positive association between overall physical activity and the risk of inguinal hernia, particularly among obese individuals.^[26]^ Interestingly, the study also observed that prolonged sedentary behavior appeared to correlate negatively with the risk of inguinal hernia.^[26]^ Conversely, other studies suggest that maintaining appropriate levels of physical activity could strengthen the abdominal wall muscles, potentially reducing the risk of hernia formation. The findings of this study indicate that MVPA is associated with a higher risk of inguinal hernia, whereas ABAA does not demonstrate a significant causal link with hernia occurrence. The authors hypothesized that prolonged periods of standing during vigorous activities may increase intra-abdominal pressure, particularly in the lower abdomen, thereby increasing the likelihood of inguinal hernia. Although this study did not identify a direct causal relationship between sedentary behavior and hernia, it is important to emphasize that the evidence for a direct causal link between sedentary behavior and hernia remains weak and inconsistent. While a potential mechanism via abdominal pressure is plausible, the current MR results do not provide robust support for this hypothesis.

The body of research on hernia remains limited, with ongoing studies primarily investigating the influence of lifestyle factors – such as smoking habits, obesity, and body fat distribution – on the occurrence of hernia. Empirical studies have demonstrated a correlation between the intensity of smoking and an increased risk of diaphragmatic hernia development.^[27]^ Furthermore, both body mass index and body fat distribution have been associated with an elevated likelihood of abdominal wall hernia.^[28–30]^ Another study also explored the causal relationship between gut microbiota and inguinal hernia, suggesting that alterations in the diversity and composition of gut microbiota may influence the incidence and outcomes of inguinal hernia.^[31]^ This study aims to fill the existing knowledge gap regarding the relationship between physical activity, sedentary behavior, and the occurrence of hernia, offering new insights for the prevention of hernia development. This suggests that improving lifestyle choices or maintaining a healthy body composition may reduce the risk of hernia formation.

This study offers valuable evidence on the causal relationships between physical activity, sedentary behavior, and the incidence of hernia, although several limitations should be acknowledged. First, despite the use of rigorously selected instrumental variables, unidentified confounding variables may still influence the accuracy of causal inferences. Second, although the data were derived from large European cohorts in the UK Biobank and FinnGen Biobank, the specific characteristics of these populations may limit the generalizability of the findings. To improve the broad applicability of these results, future studies should include a more diverse range of racial and regional populations.

In conclusion, this study employed a two-sample MR approach to examine the causal relationships between physical activity, sedentary behavior, and hernia occurrence. The findings suggest that engaging in MVPA may be associated with an increased risk of inguinal hernia, indicating that caution should be advised regarding high-intensity activities in hernia prevention rather than a general recommendation for physical activity. Nevertheless, this study has some limitations, and further research is required to investigate these causal relationships across different populations.

## Acknowledgments

The authors sincerely thank all the researchers associated with the FinnGen Biobank and UK Biobank for making their data publicly accessible. The authors also acknowledge the MRC Integrated Epidemiology Unit (IEU) at the University of Bristol for their contributions in developing the GWAS public database.

## Author contributions

**Conceptualization:** Qian Yu, Guodong Song.

**Formal analysis:** Qian Yu.

**Resources:** Qian Yu, Rujun Zhai.

**Writing – original draft:** Qian Yu, Li Wang.

**Methodology:** Li Wang.

**Data curation:** Rujun Zhai.

**Software:** Rujun Zhai.

**Validation:** Rujun Zhai.

**Visualization:** Rujun Zhai.

**Supervision:** Guodong Song.

**Writing – review & editing:** Guodong Song.










