# Supplementary material for: Investigating the causal effects of physical activity and sedentary behavior on hernia risk: A two-sample Mendelian randomization approach
Source: Medicine (Baltimore). 2026 Sep 4;105(36):e50555. doi: 10.1097/MD.0000000000050555 (PMC13549641; doi:10.1097/MD.0000000000050555)
Supplement: Supplementary file 1 [file medi-105-e50555-s001.docx]

**Table S1** Relevant snps used in the study

| MVPA | | | | | | | |
| --- | --- | --- | --- | --- | --- | --- | --- |
| SNP | EA | NEA | EAF | Beta | SE | P value | F-statistic |
| rs10145335 | A | G | 0.251 | 0.014 | 2.54E-03 | 2.70E-08 | 30.878 |
| rs1043595 | A | G | 0.283 | -0.014 | 2.45E-03 | 4.30E-09 | 34.481 |
| rs1186721 | A | G | 0.316 | 0.013 | 2.37E-03 | 4.40E-08 | 29.984 |
| rs12912808 | T | C | 0.149 | -0.018 | 3.11E-03 | 1.70E-08 | 31.853 |
| rs1921981 | A | G | 0.326 | -0.013 | 2.37E-03 | 3.80E-08 | 30.224 |
| rs1972763 | T | C | 0.658 | -0.013 | 2.32E-03 | 3.30E-08 | 30.526 |
| rs1974771 | A | G | 0.100 | 0.021 | 3.68E-03 | 6.60E-09 | 33.654 |
| rs2035562 | G | A | 0.672 | 0.014 | 2.36E-03 | 3.90E-09 | 34.688 |
| rs2114286 | G | A | 0.534 | 0.012 | 2.22E-03 | 3.30E-08 | 30.501 |
| rs2942127 | A | G | 0.825 | -0.016 | 2.90E-03 | 3.30E-08 | 30.522 |
| rs2988004 | G | T | 0.442 | 0.013 | 2.24E-03 | 4.10E-09 | 34.579 |
| rs429358 | C | T | 0.154 | 0.022 | 3.05E-03 | 6.10E-13 | 51.824 |
| rs4886868 | G | T | 0.586 | 0.012 | 2.27E-03 | 3.50E-08 | 30.405 |
| rs7326482 | T | G | 0.615 | 0.013 | 2.29E-03 | 1.60E-08 | 31.915 |
| rs77742115 | C | T | 0.138 | 0.018 | 3.20E-03 | 9.60E-09 | 32.922 |
| rs7804463 | C | T | 0.470 | -0.015 | 2.21E-03 | 1.20E-11 | 45.988 |
| rs877483 | C | T | 0.567 | -0.012 | 2.23E-03 | 4.00E-08 | 30.132 |
| rs921915 | C | T | 0.588 | 0.014 | 2.24E-03 | 5.70E-10 | 38.437 |
| ABAA | | | | | | | |
| SNP | EA | NEA | EAF | Beta | SE | P value | F-statistic |
| rs11012732 | G | A | 0.332 | -0.225 | 3.856E-02 | 5.40E-09 | 34.036 |
| rs12522261 | A | G | 0.343 | -0.211 | 3.830E-02 | 3.90E-08 | 30.207 |
| rs148193266 | C | A | 0.043 | 0.510 | 9.215E-02 | 3.10E-08 | 30.671 |
| rs34517439 | A | C | 0.121 | -0.308 | 5.624E-02 | 4.40E-08 | 29.972 |
| rs56194509 | G | T | 0.220 | 0.303 | 4.394E-02 | 5.00E-12 | 47.677 |
| rs59499656 | T | A | 0.344 | 0.228 | 3.826E-02 | 2.40E-09 | 35.598 |
| rs6775319 | T | A | 0.729 | -0.225 | 4.080E-02 | 3.50E-08 | 30.431 |
| rs9293503 | C | T | 0.112 | -0.329 | 5.869E-02 | 2.10E-08 | 31.420 |
| Television watching | | | | | | | |
| SNP | EA | NEA | EAF | Beta | SE | P value | F-statistic |
| rs10109061 | G | A | 0.443 | -0.009 | 1.62E-03 | 3.10E-08 | 30.672 |
| rs10189857 | G | A | 0.432 | 0.015 | 1.61E-03 | 5.70E-20 | 83.718 |
| rs10269099 | T | G | 0.391 | 0.009 | 1.63E-03 | 2.10E-08 | 31.412 |
| rs10739499 | G | C | 0.663 | -0.009 | 1.68E-03 | 4.20E-08 | 30.050 |
| rs10765777 | C | A | 0.392 | -0.011 | 1.64E-03 | 4.90E-12 | 47.730 |
| rs11191129 | T | C | 0.423 | -0.010 | 1.61E-03 | 9.30E-10 | 37.466 |
| rs11222919 | G | T | 0.175 | -0.012 | 2.10E-03 | 6.30E-09 | 33.750 |
| rs11245482 | C | T | 0.385 | 0.010 | 1.64E-03 | 4.80E-10 | 38.772 |
| rs114600294 | C | G | 0.208 | 0.011 | 1.96E-03 | 3.10E-08 | 30.625 |
| rs114755463 | A | G | 0.168 | 0.013 | 2.15E-03 | 6.70E-10 | 38.097 |
| rs115608101 | T | C | 0.130 | -0.014 | 2.37E-03 | 1.10E-08 | 32.599 |
| rs11662211 | T | C | 0.506 | -0.009 | 1.59E-03 | 2.90E-09 | 35.243 |
| rs11680095 | T | C | 0.593 | -0.009 | 1.63E-03 | 1.90E-08 | 31.619 |
| rs11696187 | T | C | 0.160 | -0.014 | 2.17E-03 | 4.20E-10 | 39.029 |
| rs11700249 | G | T | 0.409 | 0.009 | 1.62E-03 | 5.90E-09 | 33.860 |
| rs11714337 | A | G | 0.430 | -0.010 | 1.61E-03 | 7.60E-10 | 37.855 |
| rs11877758 | G | T | 0.313 | 0.011 | 1.72E-03 | 1.30E-10 | 41.285 |
| rs11911112 | C | A | 0.366 | -0.011 | 1.65E-03 | 8.00E-11 | 42.248 |
| rs12045585 | A | G | 0.131 | -0.015 | 2.41E-03 | 4.10E-10 | 39.068 |
| rs12214364 | G | T | 0.415 | 0.009 | 1.65E-03 | 3.80E-08 | 30.234 |
| rs12553324 | G | C | 0.415 | -0.014 | 1.62E-03 | 4.50E-17 | 70.524 |
| rs1291871 | C | T | 0.514 | 0.009 | 1.60E-03 | 9.90E-09 | 32.862 |
| rs13014947 | A | G | 0.575 | 0.011 | 1.62E-03 | 6.50E-11 | 42.663 |
| rs13107325 | T | C | 0.075 | 0.020 | 3.02E-03 | 1.70E-11 | 45.327 |
| rs1324491 | A | G | 0.131 | 0.013 | 2.36E-03 | 1.30E-08 | 32.370 |
| rs1727332 | T | C | 0.754 | 0.013 | 1.85E-03 | 1.10E-12 | 50.601 |
| rs17789218 | C | T | 0.244 | -0.011 | 1.85E-03 | 3.60E-09 | 34.845 |
| rs178203 | C | T | 0.750 | 0.012 | 1.84E-03 | 5.90E-11 | 42.845 |
| rs180396 | T | C | 0.747 | 0.010 | 1.83E-03 | 4.30E-08 | 30.007 |
| rs1826510 | A | G | 0.828 | -0.013 | 2.26E-03 | 1.90E-08 | 31.621 |
| rs1889996 | G | T | 0.739 | 0.012 | 1.81E-03 | 3.90E-11 | 43.664 |
| rs1993092 | C | T | 0.390 | -0.010 | 1.63E-03 | 5.40E-09 | 34.051 |
| rs2073869 | T | C | 0.167 | -0.014 | 2.14E-03 | 1.10E-10 | 41.611 |
| rs2106164 | C | T | 0.532 | -0.009 | 1.60E-03 | 4.20E-09 | 34.513 |
| rs2185490 | C | A | 0.618 | 0.009 | 1.64E-03 | 1.20E-08 | 32.471 |
| rs2240857 | G | T | 0.141 | 0.016 | 2.30E-03 | 4.50E-12 | 47.890 |
| rs2283 | G | A | 0.339 | -0.009 | 1.68E-03 | 3.20E-08 | 30.592 |
| rs2332818 | C | T | 0.731 | 0.010 | 1.80E-03 | 4.00E-08 | 30.172 |
| rs2352984 | C | T | 0.431 | 0.020 | 1.60E-03 | 7.40E-35 | 151.703 |
| rs2479968 | G | A | 0.051 | 0.021 | 3.70E-03 | 2.70E-08 | 30.922 |
| rs249960 | G | A | 0.182 | -0.012 | 2.07E-03 | 1.10E-08 | 32.633 |
| rs262890 | G | A | 0.299 | 0.013 | 1.74E-03 | 2.60E-14 | 58.042 |
| rs263771 | A | C | 0.233 | 0.012 | 1.89E-03 | 1.90E-10 | 40.602 |
| rs2646351 | A | G | 0.453 | 0.009 | 1.60E-03 | 2.40E-08 | 31.101 |
| rs2678662 | G | T | 0.608 | 0.011 | 1.63E-03 | 8.60E-12 | 46.621 |
| rs2725371 | G | A | 0.696 | -0.013 | 1.73E-03 | 2.10E-14 | 58.479 |
| rs2857693 | T | G | 0.366 | 0.011 | 1.65E-03 | 9.60E-11 | 41.907 |
| rs2906604 | C | T | 0.497 | 0.010 | 1.59E-03 | 5.50E-11 | 42.994 |
| rs3138499 | C | A | 0.518 | 0.011 | 1.61E-03 | 8.50E-12 | 46.658 |
| rs34094119 | G | A | 0.525 | 0.009 | 1.60E-03 | 1.30E-08 | 32.382 |
| rs34811474 | A | G | 0.231 | -0.013 | 1.89E-03 | 3.10E-12 | 48.598 |
| rs35797019 | G | A | 0.392 | -0.009 | 1.63E-03 | 1.50E-08 | 32.040 |
| rs362312 | C | T | 0.424 | -0.010 | 1.61E-03 | 3.50E-09 | 34.893 |
| rs3754970 | C | T | 0.503 | 0.010 | 1.60E-03 | 1.30E-09 | 36.828 |
| rs3810496 | C | T | 0.616 | 0.009 | 1.64E-03 | 1.20E-08 | 32.483 |
| rs4076457 | T | C | 0.257 | -0.010 | 1.82E-03 | 3.70E-08 | 30.299 |
| rs4110177 | A | G | 0.367 | 0.009 | 1.66E-03 | 1.30E-08 | 32.375 |
| rs4303732 | C | T | 0.402 | -0.011 | 1.62E-03 | 3.70E-11 | 43.779 |
| rs4339469 | G | T | 0.629 | 0.013 | 1.65E-03 | 2.80E-14 | 57.861 |
| rs4469687 | G | A | 0.484 | 0.009 | 1.59E-03 | 3.10E-08 | 30.621 |
| rs4567133 | A | C | 0.810 | -0.013 | 2.04E-03 | 1.70E-10 | 40.822 |
| rs4747438 | T | C | 0.677 | -0.012 | 1.70E-03 | 3.90E-12 | 48.152 |
| rs4788616 | G | T | 0.391 | -0.010 | 1.63E-03 | 4.30E-10 | 38.982 |
| rs4847408 | C | G | 0.649 | -0.010 | 1.66E-03 | 4.10E-10 | 39.068 |
| rs494566 | T | C | 0.332 | 0.010 | 1.70E-03 | 2.00E-09 | 35.930 |
| rs57555420 | T | C | 0.282 | 0.010 | 1.77E-03 | 9.40E-09 | 32.957 |
| rs6102912 | C | T | 0.410 | -0.011 | 1.62E-03 | 1.40E-11 | 45.603 |
| rs61743199 | G | A | 0.072 | 0.017 | 3.08E-03 | 1.60E-08 | 31.874 |
| rs61864793 | C | T | 0.252 | -0.011 | 1.83E-03 | 1.20E-09 | 36.894 |
| rs62145951 | C | T | 0.263 | -0.012 | 1.80E-03 | 2.60E-11 | 44.433 |
| rs62199883 | A | C | 0.486 | 0.014 | 1.59E-03 | 3.20E-18 | 75.768 |
| rs6493583 | G | C | 0.138 | 0.013 | 2.33E-03 | 9.50E-09 | 32.936 |
| rs6511708 | C | T | 0.665 | -0.012 | 1.69E-03 | 7.50E-13 | 51.410 |
| rs68056254 | T | G | 0.151 | 0.013 | 2.22E-03 | 1.30E-09 | 36.792 |
| rs6814554 | A | G | 0.474 | 0.014 | 1.60E-03 | 2.40E-17 | 71.823 |
| rs6850494 | C | A | 0.386 | 0.010 | 1.63E-03 | 3.40E-09 | 34.938 |
| rs6895658 | C | T | 0.193 | -0.013 | 2.01E-03 | 1.10E-10 | 41.583 |
| rs6994132 | C | T | 0.579 | -0.010 | 1.61E-03 | 6.20E-10 | 38.250 |
| rs7089973 | A | C | 0.380 | 0.009 | 1.65E-03 | 1.60E-08 | 31.892 |
| rs71658797 | A | T | 0.121 | 0.016 | 2.44E-03 | 1.10E-10 | 41.696 |
| rs7184800 | A | G | 0.303 | -0.013 | 1.73E-03 | 7.40E-15 | 60.476 |
| rs72673939 | C | G | 0.183 | 0.012 | 2.06E-03 | 4.10E-09 | 34.557 |
| rs73571431 | T | C | 0.110 | 0.016 | 2.55E-03 | 5.50E-10 | 38.496 |
| rs73946726 | A | C | 0.020 | 0.032 | 5.71E-03 | 2.20E-08 | 31.349 |
| rs749056 | G | T | 0.304 | -0.010 | 1.73E-03 | 7.20E-09 | 33.482 |
| rs749671 | A | G | 0.372 | -0.011 | 1.64E-03 | 1.60E-11 | 45.388 |
| rs7539775 | A | G | 0.743 | 0.010 | 1.82E-03 | 4.30E-08 | 30.005 |
| rs75499503 | T | C | 0.220 | -0.018 | 1.95E-03 | 3.10E-21 | 89.463 |
| rs75641275 | C | A | 0.143 | 0.015 | 2.27E-03 | 1.00E-11 | 46.239 |
| rs77273138 | A | T | 0.045 | 0.023 | 3.84E-03 | 9.70E-10 | 37.379 |
| rs7798292 | A | G | 0.435 | -0.010 | 1.60E-03 | 1.30E-09 | 36.764 |
| rs78227853 | T | C | 0.025 | -0.028 | 5.14E-03 | 3.40E-08 | 30.453 |
| rs7899206 | G | T | 0.492 | -0.010 | 1.61E-03 | 1.30E-09 | 36.852 |
| rs7921305 | A | G | 0.253 | -0.011 | 1.83E-03 | 3.70E-10 | 39.257 |
| rs79373894 | C | T | 0.034 | -0.029 | 4.44E-03 | 9.80E-11 | 41.862 |
| rs801733 | C | A | 0.358 | -0.012 | 1.66E-03 | 5.00E-14 | 56.723 |
| rs814197 | G | T | 0.467 | -0.011 | 1.59E-03 | 2.00E-11 | 44.948 |
| rs872169 | G | C | 0.206 | 0.011 | 1.97E-03 | 8.20E-09 | 33.233 |
| rs883027 | C | G | 0.420 | 0.010 | 1.61E-03 | 2.00E-09 | 36.016 |
| rs898751 | T | C | 0.493 | 0.010 | 1.59E-03 | 3.00E-10 | 39.664 |
| rs9300594 | G | A | 0.254 | 0.011 | 1.83E-03 | 9.10E-10 | 37.505 |
| rs9326862 | T | A | 0.531 | -0.009 | 1.59E-03 | 6.80E-09 | 33.595 |
| rs9471333 | T | C | 0.552 | -0.010 | 1.60E-03 | 6.70E-11 | 42.592 |
| rs9834970 | C | T | 0.498 | -0.009 | 1.59E-03 | 2.00E-08 | 31.491 |
| rs9867121 | A | C | 0.184 | -0.012 | 2.06E-03 | 1.40E-08 | 32.239 |
| rs9867437 | C | A | 0.460 | -0.010 | 1.60E-03 | 1.30E-10 | 41.366 |
| rs9880023 | T | G | 0.556 | 0.010 | 1.61E-03 | 2.00E-09 | 35.994 |
| rs996234 | A | G | 0.516 | -0.011 | 1.65E-03 | 1.60E-10 | 40.916 |
| Computer use | | | | | | | |
| SNP | EA | NEA | EAF | Beta | SE | P value | F-statistic |
| rs10208088 | T | C | 0.580 | -0.010 | 1.85E-03 | 3.00E-08 | 30.685 |
| rs1037091 | T | C | 0.327 | -0.015 | 1.98E-03 | 5.90E-15 | 60.918 |
| rs10518019 | G | A | 0.476 | 0.010 | 1.82E-03 | 4.90E-08 | 29.738 |
| rs10828248 | G | A | 0.345 | 0.011 | 1.92E-03 | 2.80E-08 | 30.846 |
| rs11259902 | A | C | 0.200 | 0.013 | 2.29E-03 | 1.60E-08 | 31.895 |
| rs112600282 | G | A | 0.110 | -0.017 | 2.92E-03 | 7.20E-09 | 33.481 |
| rs113851275 | A | G | 0.108 | 0.019 | 2.94E-03 | 2.50E-10 | 39.994 |
| rs11634155 | C | T | 0.335 | -0.012 | 1.95E-03 | 2.50E-09 | 35.556 |
| rs11652437 | A | C | 0.324 | 0.013 | 1.98E-03 | 1.00E-10 | 41.773 |
| rs117405403 | C | G | 0.585 | 0.012 | 1.85E-03 | 3.20E-10 | 39.557 |
| rs11749912 | G | A | 0.575 | -0.013 | 1.85E-03 | 3.70E-12 | 48.256 |
| rs11942953 | C | T | 0.537 | -0.010 | 1.84E-03 | 1.30E-08 | 32.324 |
| rs12128707 | G | A | 0.264 | 0.012 | 2.08E-03 | 7.10E-09 | 33.518 |
| rs12145677 | A | G | 0.297 | 0.016 | 2.00E-03 | 6.40E-15 | 60.762 |
| rs1229984 | C | T | 0.973 | -0.034 | 5.52E-03 | 4.40E-10 | 38.911 |
| rs12521638 | G | A | 0.447 | 0.010 | 1.84E-03 | 4.20E-08 | 30.056 |
| rs12553324 | G | C | 0.416 | 0.017 | 1.85E-03 | 3.90E-21 | 89.026 |
| rs12706626 | A | G | 0.384 | 0.010 | 1.88E-03 | 2.90E-08 | 30.762 |
| rs12820967 | C | T | 0.322 | 0.012 | 1.97E-03 | 1.50E-09 | 36.557 |
| rs12946454 | T | A | 0.268 | -0.012 | 2.06E-03 | 3.10E-09 | 35.124 |
| rs13262595 | G | A | 0.561 | 0.016 | 1.84E-03 | 1.80E-17 | 72.371 |
| rs13422733 | T | C | 0.126 | -0.015 | 2.76E-03 | 4.10E-08 | 30.115 |
| rs136553 | T | C | 0.377 | 0.011 | 1.89E-03 | 2.00E-09 | 35.994 |
| rs1395020 | A | G | 0.303 | -0.011 | 1.99E-03 | 3.40E-08 | 30.466 |
| rs1448355 | T | C | 0.618 | 0.012 | 1.89E-03 | 8.00E-11 | 42.263 |
| rs1469249 | A | G | 0.211 | -0.013 | 2.25E-03 | 8.00E-09 | 33.277 |
| rs147543875 | T | C | 0.023 | -0.036 | 6.42E-03 | 1.50E-08 | 31.993 |
| rs1648906 | A | G | 0.321 | -0.011 | 1.97E-03 | 2.10E-08 | 31.428 |
| rs166835 | T | C | 0.556 | -0.011 | 1.84E-03 | 4.30E-09 | 34.481 |
| rs16912540 | G | A | 0.137 | -0.016 | 2.66E-03 | 2.70E-09 | 35.363 |
| rs17167210 | A | G | 0.436 | -0.011 | 1.84E-03 | 6.00E-10 | 38.307 |
| rs17789218 | C | T | 0.245 | 0.013 | 2.12E-03 | 2.10E-09 | 35.872 |
| rs17862355 | G | T | 0.442 | -0.011 | 1.84E-03 | 1.00E-09 | 37.243 |
| rs1987942 | C | T | 0.617 | -0.011 | 1.90E-03 | 5.40E-09 | 34.047 |
| rs1999244 | T | A | 0.220 | 0.013 | 2.21E-03 | 3.20E-09 | 35.065 |
| rs2032780 | C | T | 0.406 | 0.012 | 1.88E-03 | 4.50E-11 | 43.374 |
| rs2041687 | G | T | 0.549 | 0.012 | 1.87E-03 | 3.80E-10 | 39.230 |
| rs2068625 | C | T | 0.698 | 0.014 | 1.99E-03 | 1.00E-12 | 50.776 |
| rs206965 | C | T | 0.791 | -0.013 | 2.24E-03 | 7.90E-09 | 33.297 |
| rs2120461 | T | C | 0.661 | 0.012 | 1.93E-03 | 2.50E-10 | 40.003 |
| rs2220599 | G | C | 0.369 | 0.012 | 1.90E-03 | 8.50E-11 | 42.140 |
| rs246723 | G | A | 0.590 | -0.011 | 1.87E-03 | 1.20E-08 | 32.502 |
| rs2588543 | T | C | 0.672 | 0.011 | 1.95E-03 | 2.20E-08 | 31.287 |
| rs2734833 | A | G | 0.607 | 0.012 | 1.87E-03 | 3.80E-11 | 43.726 |
| rs2748985 | C | T | 0.544 | 0.012 | 1.83E-03 | 9.40E-12 | 46.447 |
| rs2761438 | G | A | 0.624 | -0.011 | 1.88E-03 | 1.70E-09 | 36.273 |
| rs28710456 | C | T | 0.490 | -0.010 | 1.83E-03 | 1.20E-08 | 32.426 |
| rs306755 | C | T | 0.475 | 0.010 | 1.83E-03 | 1.40E-08 | 32.179 |
| rs34238696 | G | A | 0.107 | -0.017 | 2.96E-03 | 1.30E-08 | 32.268 |
| rs3730399 | G | A | 0.064 | -0.023 | 3.72E-03 | 9.10E-10 | 37.507 |
| rs4704043 | T | C | 0.714 | 0.012 | 2.02E-03 | 8.90E-09 | 33.075 |
| rs4852252 | C | T | 0.564 | 0.010 | 1.84E-03 | 3.00E-08 | 30.718 |
| rs56229818 | C | T | 0.484 | -0.010 | 1.83E-03 | 3.00E-08 | 30.715 |
| rs58638214 | T | C | 0.398 | -0.013 | 1.87E-03 | 6.70E-12 | 47.117 |
| rs6028090 | A | G | 0.555 | 0.013 | 1.85E-03 | 1.30E-11 | 45.870 |
| rs613872 | T | G | 0.827 | -0.016 | 2.41E-03 | 7.70E-11 | 42.330 |
| rs6449708 | C | T | 0.532 | -0.011 | 1.83E-03 | 7.90E-09 | 33.302 |
| rs6780848 | G | T | 0.270 | 0.011 | 2.06E-03 | 3.30E-08 | 30.542 |
| rs6935828 | T | C | 0.556 | 0.010 | 1.84E-03 | 3.10E-08 | 30.650 |
| rs7020477 | G | A | 0.267 | -0.012 | 2.07E-03 | 7.00E-09 | 33.548 |
| rs707926 | A | G | 0.150 | 0.015 | 2.55E-03 | 1.20E-08 | 32.544 |
| rs7209653 | C | T | 0.295 | -0.013 | 2.00E-03 | 2.20E-11 | 44.772 |
| rs72828532 | C | T | 0.179 | 0.016 | 2.38E-03 | 4.50E-11 | 43.367 |
| rs72847500 | C | T | 0.121 | 0.016 | 2.81E-03 | 1.90E-08 | 31.623 |
| rs7288455 | G | A | 0.567 | -0.011 | 1.85E-03 | 1.10E-08 | 32.647 |
| rs73578186 | T | C | 0.324 | -0.012 | 1.96E-03 | 3.70E-10 | 39.265 |
| rs7526112 | G | T | 0.362 | -0.011 | 1.90E-03 | 1.10E-08 | 32.653 |
| rs75550998 | T | G | 0.054 | -0.022 | 4.10E-03 | 4.60E-08 | 29.893 |
| rs7564844 | A | G | 0.701 | -0.012 | 1.99E-03 | 5.60E-10 | 38.453 |
| rs76112266 | G | C | 0.166 | -0.016 | 2.45E-03 | 3.50E-11 | 43.862 |
| rs7630869 | T | C | 0.304 | 0.016 | 1.98E-03 | 4.00E-16 | 66.249 |
| rs7904398 | T | C | 0.503 | -0.010 | 1.83E-03 | 2.40E-08 | 31.136 |
| rs7968738 | A | G | 0.264 | -0.012 | 2.08E-03 | 2.40E-09 | 35.659 |
| rs79720045 | C | T | 0.401 | -0.013 | 1.90E-03 | 1.80E-11 | 45.231 |
| rs806795 | A | G | 0.470 | 0.010 | 1.82E-03 | 1.40E-08 | 32.230 |
| rs9375188 | T | C | 0.484 | 0.016 | 1.83E-03 | 5.00E-18 | 74.896 |
| rs9537571 | A | G | 0.096 | 0.019 | 3.11E-03 | 1.10E-09 | 37.220 |
| Driving | | | | | | | |
| SNP | EA | NEA | EAF | Beta | SE | P value | F-statistic |
| rs11057408 | T | G | 0.334 | -0.011 | 1.98E-03 | 1.20E-08 | 32.530 |
| rs12069474 | C | A | 0.219 | 0.012 | 2.27E-03 | 3.50E-08 | 30.381 |
| rs12921753 | T | C | 0.400 | 0.012 | 1.91E-03 | 2.90E-10 | 39.761 |
| rs2090660 | T | C | 0.194 | -0.013 | 2.38E-03 | 3.70E-08 | 30.311 |
| rs2426132 | C | G | 0.459 | -0.012 | 1.88E-03 | 6.90E-10 | 38.050 |
| rs2588917 | A | C | 0.448 | -0.010 | 1.89E-03 | 3.40E-08 | 30.477 |
| rs975303 | G | A | 0.181 | 0.015 | 2.44E-03 | 1.30E-09 | 36.757 |
