# Supplementary material for: Investigating the causal effects of physical activity and sedentary behavior on hernia risk: A two-sample Mendelian randomization approach
Source: Medicine (Baltimore). 2026 Sep 4;105(36):e50555. doi: 10.1097/MD.0000000000050555 (PMC13549641; doi:10.1097/MD.0000000000050555)
Supplement: Supplementary file 2 [file medi-105-e50555-s002.docx]

**Table S2** Sensitivity analysis of the causal associations between physical activity, sedentary behavior and hernia

|  |  | Heterogeneity test | | | | Pleiotropic test | | |
| --- | --- | --- | --- | --- | --- | --- | --- | --- |
| Exposure | Outcome | Method | Q | Q_df | P | Egger intercept | se | P |
| MVPA | Hernia | MR Egger | 31.145 | 16 | 0.013 | 0.007 | 0.021 | 0.730 |
|  |  | IVW | 31.385 | 17 | 0.018 |  |  |  |
|  | Inguinal Hernia | MR Egger | 15.452 | 16 | 0.492 | -0.009 | 0.019 | 0.654 |
|  |  | IVW | 15.661 | 17 | 0.548 |  |  |  |
|  | Diaphragmatic Hernia | MR Egger | 26.248 | 16 | 0.051 | 0.038 | 0.038 | 0.338 |
|  |  | IVW | 27.851 | 17 | 0.047 |  |  |  |
|  | Femoral Hernia | MR Egger | 15.611 | 16 | 0.480 | -0.036 | 0.081 | 0.663 |
|  |  | IVW | 15.808 | 17 | 0.537 |  |  |  |
|  | Umbilical Hernia | MR Egger | 19.490 | 16 | 0.244 | -0.009 | 0.037 | 0.809 |
|  |  | IVW | 19.564 | 17 | 0.297 |  |  |  |
|  | Unspecified Abdominal Hernia | MR Egger | 12.513 | 16 | 0.708 | -0.023 | 0.094 | 0.811 |
|  |  | IVW | 12.572 | 17 | 0.764 |  |  |  |
| ABAA | Hernia | MR Egger | 20.182 | 6 | 0.003 | 0.025 | 0.038 | 0.532 |
|  |  | IVW | 21.663 | 7 | 0.003 |  |  |  |
|  | Inguinal Hernia | MR Egger | 15.917 | 6 | 0.014 | 0.039 | 0.043 | 0.401 |
|  |  | IVW | 18.087 | 7 | 0.012 |  |  |  |
|  | Diaphragmatic Hernia | MR Egger | 2.635 | 6 | 0.853 | -0.009 | 0.041 | 0.838 |
|  |  | IVW | 2.680 | 7 | 0.913 |  |  |  |
|  | Femoral Hernia | MR Egger | 2.183 | 6 | 0.902 | -0.100 | 0.113 | 0.410 |
|  |  | IVW | 2.966 | 7 | 0.888 |  |  |  |
|  | Umbilical Hernia | MR Egger | 10.206 | 6 | 0.116 | -0.006 | 0.062 | 0.922 |
|  |  | IVW | 10.224 | 7 | 0.176 |  |  |  |
|  | Unspecified Abdominal Hernia | MR Egger | 8.408 | 6 | 0.210 | -0.171 | 0.154 | 0.310 |
|  |  | IVW | 10.132 | 7 | 0.181 |  |  |  |
| Television watching | Hernia | MR Egger | 88.916 | 103 | 0.837 | 0.003 | 0.006 | 0.545 |
|  |  | IVW | 89.285 | 104 | 0.848 |  |  |  |
|  | Inguinal Hernia | MR Egger | 117.304 | 103 | 0.159 | 0.009 | 0.007 | 0.207 |
|  |  | IVW | 119.138 | 104 | 0.147 |  |  |  |
|  | Diaphragmatic Hernia | MR Egger | 101.951 | 103 | 0.511 | 0.013 | 0.011 | 0.238 |
|  |  | IVW | 103.359 | 104 | 0.499 |  |  |  |
|  | Femoral Hernia | MR Egger | 97.741 | 103 | 0.628 | 0.001 | 0.030 | 0.985 |
|  |  | IVW | 97.741 | 104 | 0.654 |  |  |  |
|  | Umbilical Hernia | MR Egger | 130.016 | 103 | 0.037 | **-0.036** | **0.014** | **0.013** |
|  |  | IVW | 138.067 | 104 | 0.014 |  |  |  |
|  | Unspecified Abdominal Hernia | MR Egger | 121.805 | 103 | 0.100 | 0.024 | 0.037 | 0.529 |
|  |  | IVW | 122.277 | 104 | 0.106 |  |  |  |
| Computer use | Hernia | MR Egger | 106.587 | 74 | 0.008 | 0.011 | 0.009 | 0.227 |
|  |  | IVW | 108.729 | 75 | 0.007 |  |  |  |
|  | Inguinal Hernia | MR Egger | 108.072 | 74 | 0.006 | 0.006 | 0.011 | 0.573 |
|  |  | IVW | 108.540 | 75 | 0.007 |  |  |  |
|  | Diaphragmatic Hernia | MR Egger | 71.720 | 74 | 0.553 | **0.035** | **0.014** | **0.019** |
|  |  | IVW | 77.450 | 75 | 0.400 |  |  |  |
|  | Femoral Hernia | MR Egger | 81.591 | 74 | 0.255 | 0.041 | 0.041 | 0.327 |
|  |  | IVW | 82.663 | 75 | 0.255 |  |  |  |
|  | Umbilical Hernia | MR Egger | 69.588 | 74 | 0.624 | -0.013 | 0.017 | 0.437 |
|  |  | IVW | 70.199 | 75 | 0.635 |  |  |  |
|  | Unspecified Abdominal Hernia | MR Egger | 104.872 | 74 | 0.011 | 0.090 | 0.054 | 0.102 |
|  |  | IVW | 108.768 | 75 | 0.007 |  |  |  |
| Driving | Hernia | MR Egger | 9.584 | 4 | 0.048 | -0.096 | 0.069 | 0.235 |
|  |  | IVW | 14.251 | 5 | 0.014 |  |  |  |
|  | Inguinal Hernia | MR Egger | 7.538 | 4 | 0.110 | -0.145 | 0.077 | 0.131 |
|  |  | IVW | 14.284 | 5 | 0.014 |  |  |  |
|  | Diaphragmatic Hernia | MR Egger | 4.436 | 4 | 0.350 | -0.006 | 0.092 | 0.949 |
|  |  | IVW | 4.441 | 5 | 0.488 |  |  |  |
|  | Femoral Hernia | MR Egger | 1.113 | 4 | 0.892 | 0.023 | 0.239 | 0.927 |
|  |  | IVW | 1.122 | 5 | 0.952 |  |  |  |
|  | Umbilical Hernia | MR Egger | 7.457 | 4 | 0.114 | 0.012 | 0.138 | 0.935 |
|  |  | IVW | 7.471 | 5 | 0.188 |  |  |  |
|  | Unspecified Abdominal Hernia | MR Egger | 5.584 | 4 | 0.232 | -0.240 | 0.328 | 0.504 |
|  |  | IVW | 6.333 | 5 | 0.275 |  |  |  |
